# Prevalence of non-communicable diseases and access to care among non-camp Syrian refugees in northern Jordan

**DOI:** 10.1186/s13031-018-0168-7

**Published:** 2018-07-11

**Authors:** Manuela Rehr, Muhammad Shoaib, Sara Ellithy, Suhib Okour, Cono Ariti, Idriss Ait-Bouziad, Paul van den Bosch, Anais Deprade, Mohammad Altarawneh, Abdel Shafei, Sadeq Gabashneh, Annick Lenglet

**Affiliations:** 1 Médecins Sans Frontières, Operational Centre Amsterdam, Amman, Jordan; 2 School of Medicine, Cardiff University, Cardiff, UK; 3 Médecins Sans Frontières, Operational Centre Amsterdam, London, UK; 4 Ministry of Health, Amman, Jordan; 5 Médecins Sans Frontières, Operational Centre Amsterdam, Amsterdam, The Netherlands

**Keywords:** Syria, Refugees, Jordan, Non-communicable diseases, Multi-morbidities, Access to health care

## Abstract

**Background:**

Tackling the high non-communicable disease (NCD) burden among Syrian refugees poses a challenge to humanitarian actors and host countries. Current response priorities are the identification and integration of key interventions for NCD care into humanitarian programs as well as sustainable financing. To provide evidence for effective NCD intervention planning, we conducted a cross-sectional survey among non-camp Syrian refugees in northern Jordan to investigate the burden and determinants for high NCDs prevalence and NCD multi-morbidities and assess the access to NCD care.

**Methods:**

We used a two-stage cluster design with 329 randomly selected clusters and eight households identified through snowball sampling. Consenting households were interviewed about self-reported NCDs, NCD service utilization, and barriers to care.

We estimated the adult prevalence of hypertension, diabetes type I/II, cardiovascular- and chronic respiratory conditions, thyroid disease and cancer and analysed the pattern of NCD multi-morbidities. We used the Cox proportional hazard model to calculate the prevalence ratios (PR) to analyse determinants for NCD prevalence and logistic regression to determine risk factors for NCD multi-morbidities by calculating odds ratios (ORs).

**Results:**

Among 8041 adults, 21.8%, (95% CI: 20.9–22.8) suffered from at least one NCD; hypertension (14.0, 95% CI: 13.2–14.8) and diabetes (9.2, 95% CI: 8.5–9.9) were the most prevalent NCDs. NCD multi-morbidities were reported by 44.7% (95% CI: 42.4–47.0) of patients. Higher age was associated with higher NCD prevalence and the risk for NCD-multi-morbidities; education was inversely associated.

Of those patients who needed NCD care, 23.0% (95% CI: 20.5–25.6) did not seek it; 61.5% (95% CI: 54.7–67.9) cited provider cost as the main barrier. An NCD medication interruption was reported by 23.1% (95% CI: 20–4-26.1) of patients with regular medication needs; predominant reason was unaffordability (63.4, 95% CI: 56.7–69.6).

**Conclusion:**

The burden of NCDs and multi-morbidities is high among Syrian refugees in northern Jordan. Elderly and those with a lower education are key target groups for NCD prevention and care, which informs NCD service planning and developing patient-centred approaches.

Important unmet needs for NCD care exist; removing the main barriers to care could include cost-reduction for medications through humanitarian pricing models. Nevertheless, it is still essential that international donors agencies and countries fulfill their commitment to support the Syrian-crisis response.

**Electronic supplementary material:**

The online version of this article (10.1186/s13031-018-0168-7) contains supplementary material, which is available to authorized users.

## Background

Since the beginning of the Syrian crisis in March 2011, more than five million people have fled Syria to neighbouring countries in the region such as Turkey, Lebanon, Jordan, Iraq and Egypt [1]. In Jordan alone, the United Nations High Commission for Refugees (UNHCR) has registered more than 650,000 Syrian refugees, 79% of whom reside outside of refugee camps and live in urban, peri-urban and rural areas within the Jordanian host communities [2].

In the Eastern Mediterranean Region (EMR), the burden of non-communicable diseases (NCDs) is high: the age-standardized mortality from NCDs in 2012 was 572.7 and 640.3 deaths per 100,000 population for Syria and Jordan, respectively, which is more than 10-fold higher than the mortality rates from communicable diseases [3]. Previous household surveys conducted among Syrian refugees estimated that about 43 and 50% of all refugee households in Jordan and Lebanon had at least one member with an NCD, including diabetes, hypertension, cardiovascular diseases, chronic respiratory diseases and/or arthritis [4, 5]. The most prevalent conditions reported among adults were arthritis and hypertension [4, 5]. Information about the prevalence and patterns of NCD multi-morbidities or determinants for high NCD prevalence in this vulnerable population are currently absent.

The high NCD burden among Syrian refugees has pushed the response to the current crisis to adapt its health interventions. Traditionally, humanitarian responses focussed on short-term control of communicable diseases, while the management of NCDs requires a long-term approach with sometimes complex and costly interventions. The need for NCD care in emergencies is increasingly recognized and essential standards, priority actions and medical guidelines for the humanitarian responses are being continuously developed and implemented [6–9]. To address the vast health care needs of the Syrian refugee population, host counties have also had to adjust their crisis responses. In Jordan, in light of the funding shortages in the recent years [10], national policies have been altered in order to strengthen national systems across all sectors, fostering economic growth and thereby addressing the needs of the Syrian refugees as well as host communities [11–13]. One policy, directly linked with access to health care, is the introduction of user-fees for Syrian refugees at public health facilities in Jordan. While previously free-of-charge, since November 2014, non-camp Syrian refugees have to pay for most health services albeit at a lower, government-subsidized rate [14].

Access to health care for refugees, including access to NCD care, was previously studied in a national, representative survey in 2014 [4], prior to the introduction of user fees in Jordan. The authors report that the majority of Syrian refugees in Jordan did seek NCD care and identified unaffordability as the main barrier to care [4, 15, 16]. Importantly, user-fees for health care have been linked with utilization and accessibility of health services in other contexts [17–19], hence more evidence is required to better understand the current health access situation of Syrian refugees in Jordan.

Among other actors, Médecins Sans Frontières (MSF) currently provides NCD care in three outpatient primary care clinics in northern Jordan, which, by May 2017 had enrolled almost 3500 Syrian refugees and 1500 vulnerable Jordanians. To inform and guide health service planning efforts for the refugee population in northern Jordan, we conducted a cross-sectional survey to estimate the prevalence of NCDs, investigate the pattern of NCD multi-morbidities and determine factors associated with high NCD prevalence. Given the recent introduction of user fees for utilization of health care services by refugees, we also used the opportunity to assess the current situation with regards to access to NCD care in the same area.

## Methods

### Study design

**Fig. 1:**
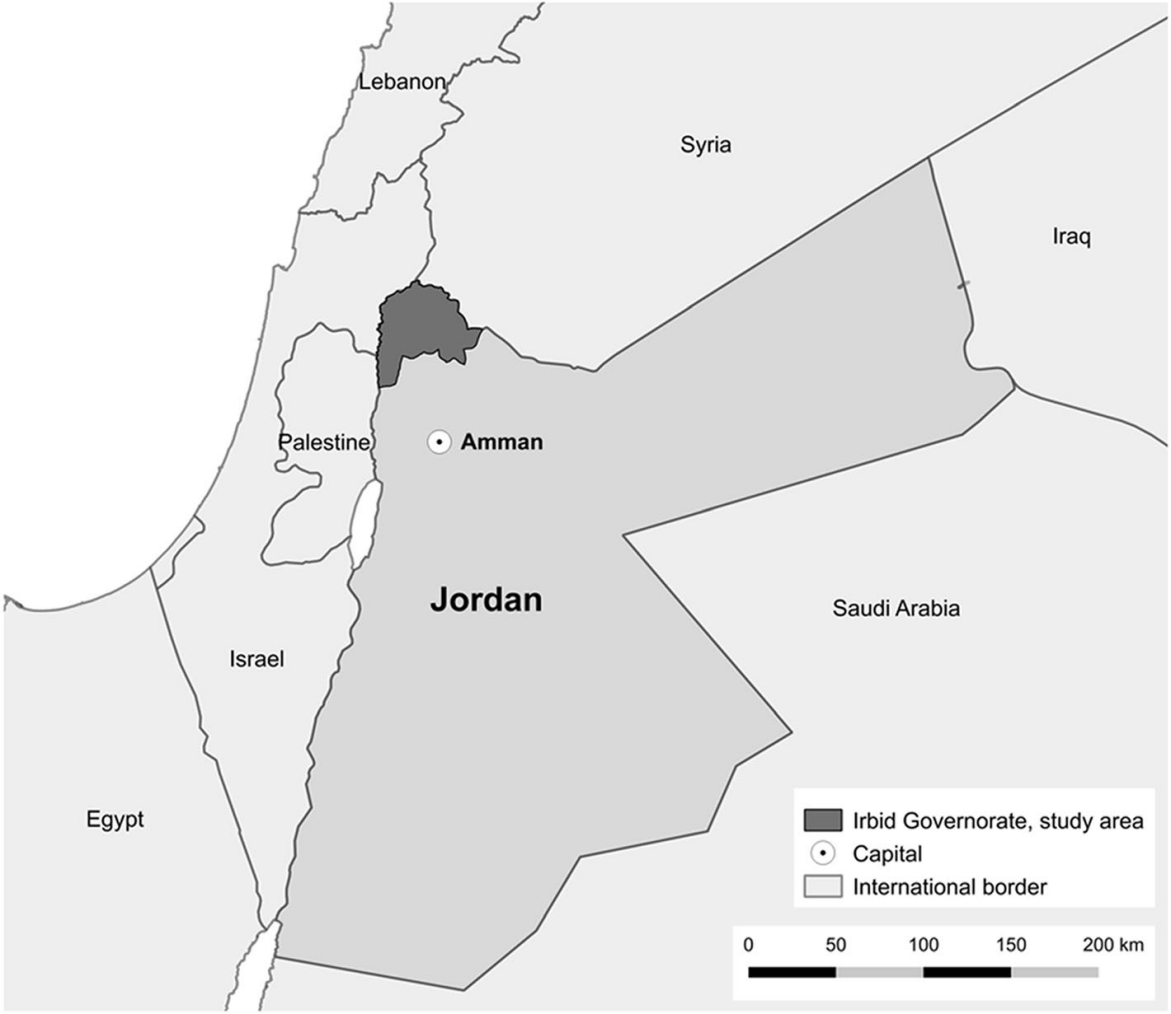
Regional map and survey area: Irbid governorate

We conducted a cross-sectional household survey in Irbid governorate in northern Jordan (Fig. 1) using two-stage cluster methodology. In the first stage, clusters were selected from inhabited areas situated in the study area using sampling with probability of allocation proportional to the respective refugee population size of each village. We used estimated population data from UNHCR-registered Syrian refugees living in Irbid governorate as of March 2016, assuming that the geographical distribution of registered and unregistered refugees did not differ.

Clusters and the starting points for household inclusion within each cluster were randomly selected Global Positioning Systems (GPS) points. These were generated using Quantum Geographic Information Systems (GIS) software (QGIS, v.2.12.1), which randomly assigned GPS points within the geographical limit of villages and cities in the governorate. As official municipal boundaries for GIS software for the governorate did not exist, we newly traced the geographical boundaries of each of the villages or cities. For the second stage we selected households using snowball sampling within each cluster (see below).

Sample size estimates were based on information from recent household surveys in Jordan [4, 15]. We assumed the NCD household prevalence of Syrian refugees in Jordan was 40%, a design effect of 2.0, a non-response rate of 10% and a mean household size of 6.5 individuals. Based on these, we calculated that 2616 households needed to be interviewed, divided over 327 clusters with eight households in each cluster.

The Ethical Review Board of MSF and the Ministry of Health in Jordan approved the study protocol.

### Participants

The study population consisted of the Syrian refugees living outside official refugee camps in Irbid governorate. Syrian households were considered for inclusion in the survey if they arrived in Jordan in or after January 2012 and if they were living in Jordan in the 6 months prior to the survey interview.

### Procedures

The survey was conducted between May 22 and June 28 2016. We used 18 interviewer teams of two data collectors (one male and one female). Teams were trained for 2.5 days on the methods of sampling, interviewing skills, the questionnaire and data collection tools. The training included a pilot test for the questionnaire.

Survey teams used tablet devices (GPS-app: OsmAnd) to locate the GPS-starting point of the respective cluster. Teams identified the first Syrian household that was geographically nearest to the cluster coordinates by asking pedestrians, shop owners or by approaching houses directly. After the first interview was completed, teams identified another seven households through referral, starting from the first interviewed household (i.e. snowball sampling). Clusters that were not accessible due to security reasons were replaced by clusters in the nearest accessible village. In areas in which data collectors interviewed all resident households but could not identify more, the clusters were cancelled and replacement clusters were randomly selected in nearby areas.

Prior to every interview, written informed consent was obtained from the head of the household. In the absence of the head of household any adult family member (≥ 18 years) could provide written informed consent in their place. The consenting respondent was asked about socio-demographics of every household member and household economics (see next section). If one adult household member was reported to suffer from an NCD, s/he was invited for a more detailed interview on access to NCD care. If there was more than one household member with an NCD, one was randomly selected using random numbers. Prior to commencing with the interview about the access to NCD care, the respondent was asked for oral informed consent, which was witnessed and noted in the questionnaire by the data collectors.

The questionnaire was formatted using the open-source toolbox Open Data Kit (ODK) [20]. It consisted of the following sections: 1) Household level data (the time of arrival in Jordan, governorate of origin in Syria, household economics); 2) Individual level data (age, gender and educational level, physical disabilities, legal registration status and presence of non-communicable diseases for every member). 3) Access to NCD care (NCD health care needs in the past 6 months, NCD care-seeking behaviour the last time care was needed, health sector utilization and expenditures the last time NCD care was sought, perceived main barriers to NCD care).

The questionnaire was designed drawing from MSFs experience in conducting similar surveys in other projects and from previously conducted surveys among Syrian refugees in Jordan [4]. Phrasing and definitions were reviewed and agreed upon with relevant technical experts in MSF and MoH. The questionnaire was initially written in English and translated into Arabic. Correctness and accuracy were checked and adjusted by Arabic-speaking experts and finally piloted among Syrian households in two villages, which were outside the survey sampling-scheme. Corrections were made after the pilot.

### Variables and definitions

A household was defined as a person or a group of people, who live together in the same unit and who are under the responsibility of the head of household. Household members were defined as those individuals living in the household at any time during the recall period (6 months prior to the survey). Collected socio-demographic data on individual household members included: age (numerical), gender (male/female), highest level of education (no education/primary/secondary/high school/university) and disability (defined as having a physical impairment that has a substantial negative effect on the persons ability to do normal daily activities).

Individuals were considered as being legally registered refugees if they reported that they were registered with UNHCR and had obtained a Ministry of Interior Card (MOI) from the Jordanian government.

Having an NCD was defined as suffering from one or more of the following conditions: hypertension, diabetes mellitus type I or II (combined), cardiovascular conditions, chronic respiratory conditions, thyroid disease and/or cancer. The presence of NCDs was self-reported and the data collectors did not ask for further medical documentation to verify the information given. NCD multi-morbidities were defined as having two or more of the six investigated NCDs. The assessment of NCD morbidities focussed on those NCDs for which MSF provides medical care in the study catchment area.

We collected information on household characteristics and economics, which included: household size (calculated from individual member data), date of arrival in Jordan, governorate of origin in Syria, household income in the month prior to the survey (defined as monetary income from cash assistance, work, family financial support, loans or others sources to be specified). Cash generated from selling World Food Program (WFP) food vouchers was excluded from monetary income and separately considered under the variable “Received WFP food vouchers in the month prior to the survey”. We also collected information on household expenditures and whether the household was in debt. Households were considered to be urban if they resided in Irbid city or Ramtha city; otherwise they were classified as rural.

We assessed access to healthcare by asking whether self-report NCD patients had required and sought NCD care in the past 6 months. We asked if the type of care sought had been accessed through the public, private or NGO sector. We also asked which direct costs (consultations, diagnostics and medications) were incurred for the last reported NCD care visit. If individuals reported that they had not sought any care for the NCD when it was required, we inquired for the reasons for this. We also asked NCD patients on their current needs for NCD medication and whether they had stopped taking NCD medication in the last 6 months for longer than 2 weeks (and the reasons for this).

### Statistical analysis

We calculated proportions of descriptive variables including their 95% confidence intervals (95% CI). Cox Proportional Hazard Regression modelling [21, 22] was used to analyse determinants for NCD prevalence including age, gender, education and location of living. The magnitude of association is presented as prevalence ratios (PRs) and 95% CI and *p*-values were calculated from Wald Tests. Logistic regression modelling was used to analyse factors associated with NCD-multi-morbidities including age, gender, education on location of living. The magnitude of association is presented as Odds ratios (ORs) and 95% CI and *p*-values were calculated from Wald Tests.

To analyse characteristics associated with NCD care-seeking behaviour logistic regression was used including the following variables: age, gender, education, type of NCDs, legal registration status, physical disabilities, household income, −debt, and -size, having received WFP food vouchers as well as location of living. The fully adjusted regression model controlled for age, gender, education, reported diabetes, hypertension, cardiovascular and respiratory conditions, household income and -size. The magnitude of association for logistic regression analyses are presented as ORs and 95% CI with respective p-values calculated from Wald Tests.

The analysis was conducted using STATA 14 (StataCorp, College Station, TX, USA). All analyses accounted for the two-stage cluster survey design by using “svyset” to declare the survey design and the svy-prefix for subsequent analyses [23].

## Results

### Characteristics of Syrian refugees

A total of 2712 households were approached of which 2587 (95.4%) consented to the interview. The majority of households (79.0%) originated from Dar’a governorate in southern Syria and had lived on average 3.42 years in Jordan at the time of the survey. The mean household expenditure exceeded the mean income (508.2 USD vs. 338.3 USD) and the majority of households reported being in debt (79.3%) (Additional file 1: Table S1).

The survey included 17,579 individuals, which comprised approximately 12.4% of the entire non-camp Syrian refugee population estimated by UNHCR for Irbid governorate at the time of the survey, including those with- and without a UNHCR Asylum Seeker Certificate [2]. Among those, 8041 (45.7%) were adults ≥18 years of age and 55.7% of adults were female. The majority of adults (79.8%) reported a fully legal status, i.e. holding a UNHCR Asylum Seeker Certificate and a MOI card. While 63.7% of adults had completed at least secondary school, 12.6% reported no formal education (Additional file 1: Table S1).

### NCD prevalence

Among adult Syrian refugees, 21.8% (95% CI 20.9–22.8) reported they suffered from at least one NCD, including hypertension, diabetes, cardiovascular- or chronic respiratory conditions, thyroid diseases or cancer. The most prevalent reported NCD was hypertension (14.0, 95% CI 13.2–14.8), followed by diabetes type I/II (9.2, 95% CI 8.5–9.9) and cardiovascular conditions (5.7, 95% CI 5.1–6.3). Chronic respiratory conditions, thyroid disease or cancer were reported by 3.2% (95% CI 2.8–3.6), 2.5% (95% CI: 2.2–2.9) and 0.6% (0.4–0.7) of adults, respectively.

### Determinants for high NCD prevalence

More women reported having hypertension compared to men (aPR 1.36, 95% CI 1.23–1.50) (Table 1). Similar observations were made for diabetes where the prevalence was higher in women than in men (aPR: 1.22, 95% CI 1.07–1.40). In contrast, the prevalence of cardiovascular- and chronic respiratory diseases were comparable in men and women; neither unadjusted nor adjusted regression analysis revealed that gender was a determinant for high prevalence (Table 1).

**Table 1:** NCD prevalence and prevalence ratios (PR). Fully adjusted PRs (aPR) controlled for all variables: age, gender, education and location of living (*N* = 8029)

		Prevalence	Unadjusted PR	Adjusted PR (aPR)	Wald Test
	n	% (95% CI)	(95% CI)	(95% CI)	*p* value
Hypertension					
Gender					
Male	394	11.1% (10.1–12.1)	reference	reference	
Female	732	16.4% (15.4–17.4)	1.50 (1.34–1.64)	1.36 (1.23–1.50)	< 0.001
Age					
18–39 years	110	2.2% (1.8–2.7)	reference	reference	
40–59 years	504	24.0% (22.2–26.0)	11.14 (8.91–13.94)	10.45 (8.36–13.06)	< 0.001
≥60 years	512	61.8% (58.5–65.1)	28.69 (23.22–35.44)	23.48 (18.87–29.23)	
Education					
None	439	43.5% (40.2–46.8)	5.74 (5.09–6.46)	1.42 (1.25–1.61)	
Primary	299	15.8% (14.1–17.6)	2.08 (1.80–2.40)	1.31 (1.15–1.49)	< 0.001
Secondary & higher	388	7.6% (6.9–8.3)	reference	reference	
Location of residence					
Rural	480	13.1% (12.0–14.3)	reference	reference	
Urban	646	14.8% (13.8–15.8)	1.12 (1.01–1.26)	1.07 (0.97–1.18)	0.170
Diabetes (type I/II)					
Gender					
Male	275	7.7% (6.9–8.7)	reference	reference	
Female	465	10.4% (9.6–11.3)	1.35 (1.18–1.53)	1.22 (1.07–1.40)	0.004
Age					
18–39 years	61	1.2% (0.9–1.5)	reference	reference	
40–59 years	347	16.5% (15.0–18.3)	13.84 (10.67–17.95)	12.92 (9.93–16.81)	< 0.001
≥60 years	332	40.1% (36.8–43.5)	33.54 (26.03–43.22)	26.89 (20.44–35.36)	
Education					
None	288	28.5% (25.7–31.5)	5.73 (4.89–6.70)	1.48 (1.24–1.77)	
Primary	197	10.4% (9.0–12.0)	2.08 (1.74–2.50)	1.31 (1.10–1.56)	< 0.001
Secondary & higher	255	5.0% (4.4–5.6)	reference	reference	
Location of residence					
Rural	314	8.6% (7.6–9.7)	reference	reference	
Urban	426	9.7% (8.9–10.7)	1.13 (0.97–1.32)	1.08 (0.94–1.25)	0.286
Cardiovascular conditions					
Gender					
Male	200	5.6% (4.9–6.5)	reference	reference	
Female	256	5.7% (5.1–6.5)	1.02 (0.86–1.21)	0.93 (0.78–1.11)	0.415
Age					
18–39 years	57	1.1% (0.9–1.5)	reference	reference	
40–59 years	165	7.9% (6.7–9.2)	7.04 (5.25–9.45)	6.71 (4.98–9.04)	< 0.001
≥60 years	234	28.3% (25.2–31.6)	25.30 (19.01–33.68)	21.44 (15.43–29.79)	
Education					
None	177	17.5% (15.2–20.1)	5.54 (4.55–6.75)	1.38 (1.07–1.77)	
Primary	117	6.2% (5.1–7.5)	1.95 (1.54–2.47)	1.23 (0.98–1.54)	0.038
Secondary & higher	162	3.2% (2.7–3.7)	reference	reference	
Location of residence					
Rural	212	5.8% (5.0–6.7)	reference	reference	
Urban	244	5.6% (4.9–6.3)	0.96 (0.79–1.17)	0.91 (0.75–1.09)	0.312
Chronic respiratory conditions					
Gender					
Male	99	2.8% (2.3–3.4)	reference	reference	
Female	158	3.5% (3.0–4.1)	1.27 (0.98–1.66)	1.19 (0.91–1.57)	0.200
Age					
18–39 years	117	2.3% (1.9–2.8)	reference	reference	
40–59 years	93	4.4% (3.6–5.4)	1.93 (1.49–2.51)	1.86 (1.43–2.43)	< 0.001
≥60 years	47	5.7% (4.4–7.3)	2.48 (1.82–3.37)	1.98 (1.35–2.91)	
Education					
None	61	6.0% (4.7–7.7)	2.15 (1.62–2.85)	1.46 (1.01–2.11)	
Primary	52	2.7% (2.0–3.7)	0.97 (0.69–1.37)	0.88 (0.62–1.23)	0.052
Secondary & higher	144	2.8% (2.4–3.3)	reference	reference	
Location of residence					
Rural	128	3.5% (2.9–4.2)	reference	reference	
Urban	129	3.0% (2.5–3.5)	0.84 (0.65–1.08)	0.84 (0.65–1.08)	0.172

The prevalence of all investigated NCDs increased with age, whereby the highest increase was observed for diabetes, hypertension and cardiovascular conditions. Specifically, the prevalence of diabetes among 18–39 year old refugees was 1.2% (95% CI 0.9–1.5) and increased to 40.1% (95% CI 36.8–43.5) in adults over 59 years of age (aPR: 26.89, 95% CI 20.44–35.36) (Table 1). Similarly, the prevalence of hypertension and cardiovascular conditions were more than 20-fold higher in adults over 59 years compared to the youngest age group (Table 1).

We further observed that the level of education was associated with the prevalence of investigated NCDs. The adjusted prevalence estimates for diabetes, hypertension, cardiovascular- and chronic respiratory conditions were between 1.4 and 1.5-times higher among adults with no education compared to those with secondary and higher education (Table 1).

### NCD multi-morbidities

The burden of NCD multi-morbidities among the Syrian refugee population was high: among all adults with at least one of the six investigated NCDs, 44.7% (95% CI 42.4–47.0) reported more than one NCD. Specifically, 30.4% (95% CI 28.3–32.4) of NCD patients suffered from two NCDs, 12.6% (95% CI 11.1–14.3) suffered from three NCDs and 1.8% (95% CI 1.2–2.5) reported more than three NCDs. Most patients suffered from hypertension only (22.7%), followed by patients who suffered from hypertension in combination with diabetes (17.6%) and patients who suffered from diabetes only (10.1%) (Additional file 2: Table S2).

The risk of having NCD multi-morbidities increased with age and was higher in patients with no or low education level (Table 2). In the adjusted analysis, patients over 59 years of age were 7.3-times more likely to suffer from NCD multi-morbidities (aOR: 7.34, 95% CI: 5.15–10.47). Similarly, patients were almost twice as likely to suffer from multi-morbidities if they had no education compared to those who completed at least completed secondary school (aOR: 1.75, 95% CI: 1.32–2.32).

**Table 2:** Risk factors for NCD multi-morbidities (*N* = 1756). Fully adjusted aPRs controlled for all variables: age, gender, education and location of living

			Odds Ratio: Having more than one NCD		
	Having one NCD only	Having more than one NCD	Unadjusted OR	Adjusted OR	Wald Test
	% (n)	% (n)	(95% CI)	(95% CI)	(p value)
Gender					
Male	59.4% (390)	40.6% (267)	reference	reference	
Female	52.9% (581)	47.1% (518)	1.30 (1.07–1.58)	1.21 (0.97–1.52)	0.094
Age					
18–39 years	83.2% (301)	16.9% (61)	reference	reference	
40–59 years	59.2% (462)	40.9% (319)	3.41 (2.50–4.65)	3.07 (2.24–4.20)	< 0.001
≥60 years	33.9% (208)	66.1% (405)	9.61 (6.96–13.26)	7.34 (5.15–10.47)	
Education					
None	37.7% (204)	62.3% (337)	3.69 (2.95–4.65)	1.75 (1.32–2.32)	
Primary	53.1% (240)	46.9% (212)	1.97 (1.55–2.51)	1.57 (1.22–2.03)	< 0.001
Secondary & higher	69.1% (527)	30.9% (236)	reference	reference	
Location of residence					
Rural	57.3% (438)	42.8% (327)	reference	reference	
Urban	53.8% (533)	46.2% (458)	1.15 (0.95–1.39)	1.14 (0.93–1.40)	0.219

### Care-seeking behaviour for non-communicable diseases

In the analysis for access to NCD care among Syrian refugees in northern Jordan, we included 1243 adult, self-reported NCD patients. Of these, 1133 (91.2, 95% CI: 89.1–92.9) reported that they had needed NCD medical care in the 6 months prior to the interview. Of those NCD patients who needed care, 23.0% (95% CI: 20.5–25.6) did not seek it the last time it was needed.

The main reason for not seeking NCD care was that the costs for the medical care were perceived as too expensive (61.5%; 95% CI 54.7–67.9) (Table 3). Other frequently mentioned reasons were related to knowledge about NCD services, such as not knowing where to go or believing it is not important, which were reported by 12.7% (95% CI 9.1–17.4). Unavailability of NCD services prevented 9.6% (95% CI 6.6–13.9) of NCD patients who needed care from seeking it (Table 3).

**Table 3:** Reasons for not seeking NCD medical care the last time it was needed (*N* = 260)

	n	% (95% CI)
Affordability of NCD services: Direct health care provider costs	160	61.5% (54.7–67.9)
Knowledge of NCD services: Did not know where to go or did not think it was important	33	12.7% (9.1–17.4)
Availability of NCD services: Inadequate service quality, service/staff not available or long waiting list	25	9.6% (6.6–13.9)
Approachability of NCD services: Lack or costs of transport or incomplete legal status	14	5.4% (3.1–9.2)
Acceptability of NCD services: Rude/rejecting staff attitude	2	0.8% (0.2–3.0)
Other reasons	20	7.7% (4.8–12.1)
Don’t know	6	2.3% (1.0–5.1)

### Factors associated with NCD care-seeking behaviour

In the adjusted regression model age, household income and having diabetes, hypertension or cardiovascular conditions were independently associated with NCD care-seeking behaviour. Patients who were older than 59 years were more likely to seek NCD care if needed compared to the youngest age group of 18–39 year old NCD patients (aOR: 1.97, 95% CI: 1.14–3.42) (Table 4). Patients with hypertension or diabetes were more likely to seek care compared to patients who suffered from other NCDs (aOR: 1.80, 95% CI: 1.26–2.57 and aOR: 2.41, 95% CI: 1.71–3.40) (Table 4).

**Table 4:** Factors associated with NCD care-seeking behaviour (*N* = 964). Fully adjusted aOR controlled for age, gender, education, diabetes, hypertension, chronic respiratory disease, cardiovascular condition, household income and household size

	Did not seek NCD care when needed	Did seek NCD care when needed	Odds Ratios (OR): Sought NCD care when needed		
	% (n)	% (n)	Unadjusted OR (95% CI)	Adjusted OR (95% CI)	*p* value (Wald test)
Age groups					
18–39	35.1% (68)	65.0% (126)	reference	reference	
40–59	23.2% (103)	76.9% (342)	1.79 (1.24–2.59)	1.16 (0.77–1.76)	0.039
≥60	14.8% (48)	85.2% (277)	3.11 (2.02–4.81)	1.97 (1.14–3.42)	
Gender					
Male	23.1% (83)	76.9% (276)	reference	reference	
Female	22.5% (136)	77.5% (469)	1.04 (0.76–1.42)	0.98 (0.69–1.38)	0.891
Education					
None	18.3% (55)	81.7% (245)	reference	reference	
Primary	23.8% (63)	76.2% (202)	0.72 (0.48–1.07)	1.07 (0.67–1.70)	0.674
Secondary and higher	25.3% (101)	74.7% (298)	0.66 (0.46–0.96)	1.20 (0.77–1.88)	
Physical disability					
No	23.5% (200)	76.6% (653)	reference	–	–
Yes	17.1% (19)	82.9% (92)	1.48 (0.84–2.63)	–	–
Legal status					
No	23.0% (41)	77.0% (137)	reference	–	–
Yes	22.7% (178)	77.4% (608)	1.02 (0.70–1.48)	–	–
Conditions					
Any NCD but diabetes (type I/II)	30.4% (161)	69.6% (369)	reference	reference	
Diabetes (type I/II)	13.4% (58)	86.6% (376)	2.83 (2.03–3.94)	2.41 (1.71–3.40)	< 0.001
Any NCD but hypertension	31.5% (107)	68.5% (233)	reference	reference	
Hypertension	18.0% (112)	82.1% (512)	2.10 (1.53–2.88)	1.80 (1.26–2.57)	0.001
Any NCD but cardiovascular conditions	21.9% (154)	78.1% (550)	reference	reference	
Cardiovascular conditions	25.0% (65)	75.0% (195)	0.84 (0.61–1.16)	0.71 (0.49–1.02)	0.062
Any NCD but chronic respiratory diseases	21.2% (176)	78.8% (654)	reference	reference	
Chronic respiratory diseases	32.1% (43)	67.9% (91)	0.57 (0.37–0.87)	1.03 (0.63–1.69)	0.904
Any NCD but cancer	22.5% (211)	77.6% (729)	reference	–	–
Cancer	33.3% (8)	66.7% (16)	0.58 (0.24–1.37)	–	–
Any NCD but thyroid disease	22.3% (189)	77.7% (658)	reference	–	–
Thyroid disease	25.6% (30)	74.4% (87)	0.83 (0.54–1.29)	–	–
Household size					
1–5 members	26.2% (68)	73.9% (192)	reference	reference	
6–10 members	23.0% (133)	77.0% (446)	1.19 (0.84–1.68)	1.17 (0.81–1.70)	0.200
11–25 members	14.4% (18)	85.6% (107)	2.11 (1.17–3.78)	1.80 (0.94–3.41)	
Income					
Lowest (first quintile)	25.3% (61)	74.7% (180)	reference	reference	
(2nd quintile)	25.3% (68)	74.7% (201)	1.00 (0.69–1.45)	0.96 (0.65–1.41)	
(3rd quintile)	23.3% (27)	76.7% (89)	1.12 (0.67–1.87)	0.99 (0.58–1.69)	0.076
(4th quintile)	23.9% (43)	76.1% (137)	1.08 (0.72–1.63)	1.00 (0.66–1.53)	
Highest (5th quintile)	12.7% (20)	87.3% (138)	2.34 (1.36–4.02)	2.07 (1.16–3.70)	
Household in debt					
No	19.6% (32)	80.4% (131)	reference	–	–
Yes	23.4% (187)	76.7% (614)	0.80 (0.53–1.22)	–	–
Household received WFP food vouchers					
No	30.4% (14)	69.6% (32)	reference	–	–
Yes	22.3% (205)	77.7% (713)	1.52 (0.81–2.85)	–	–
Location of household					
Rural	23.0% (97)	77.0% (324)	reference	–	–
Urban	22.5% (122)	77.5% (421)	1.03 (0.76–1.41)	–	–

Among socio-economic characteristics of patients and their households, the monthly household income was independently associated with NCD care-seeking behaviour. NCD patients who lived in a household of the richest income quintile were twice as likely to seek NCD care compared to patients from households in the lowest income quintile (aOR: 2.07, 95% CI: 1.16–3.70) (Table 4). Other socio-economic factors such as the household debt or receiving WFP food vouchers did not show any association with NCD care-seeking behaviour in the unadjusted and adjusted analysis. There was also little evidence that education level, urban/rural living or legal registration status were associated with care-seeking behaviour (Table 4).

### Health facility utilization and expenditures for NCD care

The majority of NCD patients in Irbid governorate sought NCD care at an NGO (51.1, 95% CI: 47.2–55.0), while 27.0% (95% CI: 23.9–30.4) went to a public sector facility and 18.0% (95% CI: 15.4–20.9) sought NCD care in the private sector (Table 5).

**Table 5:** Health sector utilization and out-of-pocket expenditures for NCD care. (a) Proportion of all NCD patients who sought care, (b) among those NCD patients who sought care in the respective sector, (c) among those NCD patients who received care in the respective sector. (Exchange rate 1.0 JOD = 1.41 USD, 12.08.2017)

	Public Sector		Private Sector		NGO		Other sectors		Unknown sector		Total	
	n	% (95% CI)	n	% (95% CI)	n	% (95% CI)	n	% (95% CI)	n	% (95% CI)	n	% (95% CI)
Sector in which NCD care was sought (a) (*N* = 873)	236	27.0% (23.9–30.4)	157	18.0% (15.4–20.9)	446	51.1% (47.2–55.0)	23	2.6% (1.6–4.2)	11	1.3% (0.7–2.3)		–
Receiving NCD care (b)	*N = 236*		*N = 157*		*N = 446*		*N = 23*		*N = 11*		*N = 873*	
Received care	224	94.9% (91.3–97.1)	152	96.8% (92.5–98.7)	438	98.2% (96.5–99.1)	12	52.2% (31.7–72.0)	5	45.5% (20.2–73.3)	831	95.2% (93.4–96.5)
Did not receive care	10	4.2% (2.3–7.7)	4	2.6% (1.0–6.7)	8	1.8% (0.9–3.5)	10	43.5% (24.6–64.5)	0	0	32	3.7% (2.5–5.3)
No answer	2	0.9% (0.2–3.3)	1	0.6% (0.01–4.4)	0	0	1	4.4% (0.6–25.8)	6	54.6% (26.7–79.8)	10	1.2% (0.6–2.1)
Payment for NCD care (c)	*N = 224*		*N = 152*		*N = 438*		*N = 12*		*N = 5*		*N = 831*	
Paid for care	154	68.8% (62.0–74.8)	127	83.6% (76.7–88.7)	32	7.3% (5.1–10.3)	2	16.7% (4.0–49.2)	2	40.0% (9.9–80.1)	317	38.2% (34.7–41.8)
Did not pay for care	64	28.6% (22.6–35.4)	25	16.5% (11.3–23.3)	401	91.6% (88.3–93.9)	9	75.0% (42.9–92.3)	2	40.0% (9.9–80.1)	501	60.3% (56.6–63.8)
No answer	6	2.7% (1.2–5.8)	0	0	5	1.1% (0.5–2.7)	1	8.3% (1.1–42.4)	1	20.0% (2.7–69.4)	13	1.6% (0.9–2.9)
Out-of-pocket expenditures for NCD care												
Among those who paid for NCD care (in USD)												
Mean (SD)	134	34.0 (79.5)	115	105.0 (229.1)	31	46.3 (152.4)	1	28.3	1	35.4	282	64.2 (167.5)
Median (IQR)		7.1 (4.2, 28.3)		42.5 (21.2, 70.7)		7.1 (5.7–18.4)		28.3		35.4		21.2 (7.1, 53.8)
Including those who did not pay for NCD care (in USD)												
Mean (SD)	198	22.9 (67.2)	140	86.3 (212.1)	432	3.2 (41.8)	10	2.8 (8.9)	3	11.7 (20.4)	783	23.2 (105.1)
Median (IQR)		4.2 (0, 14.2)		28.3 (9.9, 70.7)		0 (0, 0)		0 (0–0)		0 (0–35.4)		0 (0, 7.1)

Of the interviewed NCD patients who reported they did seek NCD care when needed (*N* = 873), the majority also received care when approaching the health facility of choice (95.2, 95% CI 93.4–96.5) (Table 5). The main reason for not receiving care was reported to be unaffordability of health service provider costs, which was mentioned by 14 out of 32 patients who did not receive care (43.8, 95% CI 28.6–60.2).

Of all NCD patients who received NCD care (*N* = 831), 38.2% (95% CI: 34.7–41.8) paid for the consultation while 60.3% (95% CI: 56.6–63.8) received free-of-charge services. Approaching the private sector required 83.6% (95% CI: 76.7–88.7) of patients to pay for services, while only a small proportion of patients paid for NCD care in an NGO facility (7.3, 95% CI: 5.1–10.3) (Table 5). Among those who did pay, the mean price across all sectors was 64.2 USD, which includes only direct health care cost but not payments made for transport to the facilities or other indirect health care costs (Table 5). These costs represented 19% of the average household income in the previous month.

Since about half of the NCD patients received free-of-charge health services at an NGO-facility in northern Jordan, the average cost burden for a household with an NCD patient differs from the aforementioned actual price for a consultation, diagnostics and medication. Including those who received free-of-charge NCD services in the cost estimates, the total mean expenditures for a consultation (incl. Diagnostic tests and medications) reduced from 64.2 USD to 23.2 USD. The latter represented 7.0% of the average household income in the previous month.

### Access to NCD medication

Among all interviewed NCD patients (*N* = 1243), 92.2% (95% CI: 90.6–93.5) reported they needed regular medication for their NCD. Of those who relied on regular medication, 23.1% (95% CI 20.4–26.1) also indicated that they experienced an interruption of medication for longer than 2 weeks in the past 6 months. The predominant reason given for the medication interruption was the costs of the medication (63.4, 95% CI 56.7–69.6).

## Discussion

We found that among adult Syrian refugees in northern Jordan, 21.8% suffer from at least one NCD; 14.0 and 9.2% reported hypertension and diabetes, respectively, which is consistent with national-level prevalence estimates for NCDs in the region. According to WHO, the crude average diabetes prevalence among adults in the EMR in 2014 was 11.1%, ranging from 4.8% in Somalia to 16.2% in Egypt. For Jordan and pre-conflict Syria (2010), diabetes was estimated among 13.1 and 10.1% of adults [24]. High blood pressure in the EMR is estimated to affect 22.0% of adults, Jordan and pre-conflict Syria reported 16.4 and 20.3% [25]. National-level data for other NCDs in Syrian populations are sparse and thus it is difficult to compare our findings for chronic cardiovascular- and respiratory conditions. Our prevalence estimates are also largely comparable with findings from a national representative survey conducted in 2014 among non-camp Syrian refugees in Jordan [4, 15].

By applying our findings to UNHCR Syrian refugee statistics for Jordan [2], we estimate that approximately 60,041 Syrian adults with at least one NCD including diabetes, hypertension, cardiovascular conditions, respiratory- and thyroid disease or cancer currently live outside the refugee camps in Jordan. Hypertension, diabetes and cardiovascular conditions cause the highest number of cases, i.e. 38,559, 25,339 and 15,699 patients.

We have also gained insight into the frequency and pattern of NCD multi-morbidities, which has to our knowledge not been reported before for this population. Almost half of the NCD patients suffered from more than one NCD, which translates into 26,838 patients among all non-camp Syrian refugees in Jordan. The high burden from NCD multi-morbidities has important implications for health service planning: It has been previously shown that NCD patients with more than one condition can require more frequent health care visits including outpatient and inpatient care and might face higher out-of-pocket expenditures, depending on the health care system [26–28]. Therefore, our findings highlight the importance for integrated health services for non-camp Syrian refugees, ideally at an easily accessible primary health care (PHC) level [29, 30]. By ensuring the capacity of health care providers to manage patients with a broad variety of different NCDs, including those with multiple conditions, patient consultations at numerous facilities could be reduced and consequently lessen the burden on the health care system as well as a potential financial burden on the patient. While not entirely avoidable, referrals to specialist practitioners, secondary or tertiary care level need to be efficient to avoid treatment delays or loss of follow-up of patients. Furthermore, strong communication and coordination between facilities ensures good quality of clinical care, e.g. by reducing adverse events and can further avoid unnecessary investigations. Due to the high NCD multi-morbidity burden, these aspects are of high priority for NCD services targeting Syrian refugees in Jordan.

Consistent with other studies, we found that the prevalence of NCDs as well as multi-morbidities increases with age [26–28]. A second important determinant for high NCD prevalence was a low level of education, which was also a risk factor for reporting NCD multi-morbidities in our study. A strong linkage between education and NCD single- and multi-morbidity has been reported previously and could be, at least partially, attributed to poorer health literacy, i.e. knowledge about behavioural risk factors for NCDs (such as smoking, physical inactivity and unhealthy diet) and/or preventive behaviour [29, 31–33]. Therefore, our findings suggest that key target groups for NCD prevention and control are the elderly and those with a lower or no education. Public health care providers need to be made aware of those most vulnerable groups in order to better target for example medical and preventative education: providing information about risk factors and preventative behaviour as well as detailed medical education will support an efficient patient self-management will avoid deterioration of conditions or the development of additional NCDs. It needs to be ensured that both, the elderly and those with a lower education are reached and messages are communicated in simple and understandable terms. Both, administrative- and infrastructural adaptations within health facilities should to be additionally considered. Among others, this can include simple registration- and referral procedures, ensuring more time for appointments or removing physical barriers to access facilities [34, 35].

The high NCD prevalence and NCD multi-morbidities pose an enormous burden for the Jordanian health care system, which also has to ensure NCD services for its own population that is similarly affected by NCDs [24, 25]. A Sector Vulnerability Assessment conducted by the Jordanian government in May 2015 revealed a shortage of health centres, hospital beds and physicians based on refugee and host population needs [11]. Additionally, a survey conducted in 2014 among Syrian refugees in Jordan revealed unmet needs concerning specialized NCD care [4, 15, 16]. In light of the global funding shortages [10], the Jordanian government adjusted its response: It adopted more developmental and national systems-strengthening strategies [11–13] and also introduced user-fees at public health facilities for Syrian refugees in Jordan at a subsidized rate [14].

Previous evidence of the impact of removing user fees for any health services is mixed: It can reduce the out of pocket expenditure by patients and is often linked to increased service utilization but the overall impact depends on the context and design the health care system [17–19]. Therefore, WHO recommends that a removal of user fees should be part of a comprehensive approach implemented together with additional other polices to avoid unintended consequences, such as a deterioration of service quality or availability [17]. While the implementation of the aforementioned national crisis-response strategies are on-going in Jordan, it could be further explored if the removal of user fees for Syrian refugees are a possibility to reduce financial stress on refugees and increase access to health care without jeopardizing the national health care financing system and/or compromising quality.

Our survey reveals that in the context of changed policies, the majority of Syrian refugees in Irbid were still able to access NCD care the last time it was needed. However, we should stress that important unmet medical needs exist since almost one quarter of NCD patients who were interviewed in this study did not seek NCD care the last time it was needed. The main reported barrier was unaffordability of provider costs.

While the affordability we report on here is perceived by interviewees, it is apparent that the financial burden of NCD care for the households is significant: the average monthly household expenditure exceeded the income, so the vast majority of households were already in debt at the time of the survey. In the subsidized public health sector, more than two-thirds of the NCD patients had to pay provider costs, which accounted for 10.0% of their average monthly income. This is substantial for NCD care, which requires regular medical consultations.

Unsurprisingly, the analysis of factors associated with seeking NCD care indeed also showed that patients from households in the highest income quintile were more likely to seek care when needed compared to NCD patients from the lowest income quintile. There was however little evidence that other economic considerations, including being in debt or the receipt of additional benefits such as WFP vouchers, were important factors associated with NCD care-seeking behaviour. These observations suggest that the economic assessment of a household was incomplete and there may be additional circumstances, which affect a household’s ability to pay for NCD services. A more comprehensive economic analysis could, for example, also look at savings or the ability to sell assets or services [36].

A survey conducted prior to the introduction of user fees reported that 84.7% of all Syrian refugees in Jordan did not seek NCD care; the estimates were higher in northern Jordan (89%) than in other regions [4, 15]. While our estimates are lower (77%), a direct comparison between these studies should be done cautiously as there were some differences in survey methodologies, i.e. geographic sampling areas and definitions of regions, the assessed NCDs and differences in definitions of locations/facilities considered for NCD care [4, 15, 16].

We also found that unaffordability was the main reason that about a quarter of NCD patients experienced an interruption of NCD medication supply in the previous 6 months. Ensuring uninterrupted medication supply is key to avoid exacerbations and complications of medical conditions for NCD patients and is thus a priority intervention to limit the risk for even more complex and/or costly treatments [6, 8]. Therefore, a detailed analysis of the main cost-drivers for NCD medications in Jordan could be undertaken to identify opportunities for cost reduction to levels that are more affordable for Syrian refugees. Building on the successful experiences of price reductions for HIV/TB medications and diagnostics [37–39], humanitarian pricing models, pooled procurements and preferential usage of generic brands could be opportunities for national and global initiatives to improve access to NCD medications for Syrian refugees as well as all NCD patients in other high-burden countries.

Because our findings on individual NCD prevalence are largely comparable with national level estimates and results from previously conducted national-level surveys among Syrian refugees [4, 15, 24, 25], we believe that our findings can be generalized to Syrian refugees in other parts of Jordan although this must be done with caution as there may be differences in NCD care-seeking behaviour across the regions in Jordan [16]. It is important to emphasise though that this does not limit the relevance of our study as Irbid governorate hosts the second largest Syrian refugee community after Amman and is a centre for humanitarian response in Jordan [2]. The sample size in this study was large and thus provides robust evidence to inform the humanitarian response in Irbid governorate.

This study had several limitations: NCD patients were selected based on self-reported conditions without further verification of medical records by the data collectors. This could lead to over-or under-reporting of NCDs. This methodology was unable to capture undiagnosed NCD patients, which could lead to an underestimation of NCD burden. Although data collectors were specifically trained to search for the nearest Syrian families and not preselect by type of house or shelter, we have not specifically attempted to track families living in informal tented settlements and thus our data might be over-reporting NCD prevalence and access to care for Syrian refugees in stable living conditions. The sampling design used snowball sampling, which can lead to a sampling bias if interviewed families preferentially referred to family or friends instead of the nearest family. However, the intra-cluster referral chain to households was often interrupted during the survey, as Syrian households were often hesitant to directly refer to another household and rather gave general directions to apartment blocks or streets. Hence the impact of clustering is assumed to be low. Due to cross-sectional study-design, unmeasured confounders might affect some results, such as determinants of NCDs or factors associated with care-seeking behaviour.

## Conclusions

In this study, we provide important evidence to inform the humanitarian response addressing the NCD burden among Syrian refugees in Jordan. The burden of NCDs is high among Syrian refugees and almost half of the NCD patients suffer from multiple NCDs. Furthermore, the elderly and those with a lower level of education should be target groups for NCD prevention, treatment and care. These findings are important considerations for NCD service capacity and for developing comprehensive and approaches targeted to individual patient needs.

About a quarter of NCD patients did either not access the medical care they needed or experienced an interruption of medication; unaffordability was the main barrier to care. To remove those barriers, several options could be explored, including waiving the user fees or reducing costs for medications through humanitarian pricing models at national or global level.

Nevertheless, while the Jordanian government implements a strategy focusing on strengthening all sectors to respond to the Syrian crisis, it is still essential that international donors agencies and countries fulfill their commitment to support the national- as well as the regional crisis response.

## Additional files

Additional file 1: **Table S1.** Socio-demographic and economic description of adult Syrian refugees (*N* = 8041). (DOCX 20 kb)

Additional file 2: **Table S2.** Most frequently reported single- and multi-morbidities among adults with at least one NCD. (*N* = 1756). (DOCX 14 kb)

## Abbreviations

(a)OR: (adjusted) Odds ratio
(a)PR: (adjusted) Prevalence ratio
GIS: Geographic information system
GPS: Global positioning system
MoH: Ministry of Health
MOI: Ministry of Interior
MSF: Médecins Sans Frontières
NCD: Non-communicable disease
NGO: Non-governmental organization
PHC: Primary Health Care
UNHCR: United Nations High Commission for Refugees
WFP: World Food Program
WHO: World Health Organization
