# Supplementary material for: Prevalence of non-communicable diseases and access to care among non-camp Syrian refugees in northern Jordan
Source: Confl Health. 2018 Jul 11;12:33. doi: 10.1186/s13031-018-0168-7 (PMC6040066; doi:10.1186/s13031-018-0168-7)
Supplement: Supplementary file 1 — Table S1. Socio-demographic and economic description of adult Syrian refugees (N = 8041). (DOCX 20 kb) [file 13031_2018_168_MOESM1_ESM.docx]

**Additional File 1**

**Table 1: Socio-demographic and economic description of adult Syrian refugees (N=8,041)**

|  | **Total adults (N=8,041)** | **Male (N=3,562)** | **Female (N=4,479)** |
| --- | --- | --- | --- |
|  | % (n) | % (n) | % (n) |
| **Age** |  |  |  |
| 18-29 years | 39.1% (3,145) | 38.8% (1,383) | 39.3% (1,762) |
| 30-39 years | 24.4% (1,965) | 24.6% (876) | 24.3% (1,089) |
| 40-49 years | 16.8% (1,348) | 17.3% (615) | 16.4% (733) |
| 50-59 years | 9.4% (753) | 9.2% (326) | 9.5% (427) |
| 60-69 years | 5.7% (457) | 5.3% (190) | 6.0% (267) |
| ≥70 years | 4.6% (373) | 4.8% (172) | 4.5% (201) |
| Mean age (SD) in years | 36.8 (15.4) | 36.8 (15.6) | 36.7 (15.3) |
| Median age (IQR) in years | 33 (24, 45) | 34 (24, 45) | 33 (25, 45) |
| **Education** |  |  |  |
| None | 12.6% (1,010) | 7.8% (279) | 16.3% (731) |
| Primary | 23.6% (1,898) | 26.4% (940) | 21.4% (958) |
| Secondary & higher | 63.7% (5,121) | 65.7% (2,341) | 62.1% (2,780) |
| No answer | 0.1% (12) | 0.06% (2) | 0.2% (10) |
| **Legal documents** |  |  |  |
| Complete legal documents (UNHCR & MOI) | 79.8% (6,416) | 78.9% (2,812) | 80.5% (3,604) |
| Incomplete legal documents | 20.0% (1,607) | 20.8% (741) | 19.3% (866) |
| No answer | 0.2% (18) | 0.3% (9) | 0.2% (9) |
| **Physical disability** |  |  |  |
| Any physical disability | 6.3% (504) | 8.8% (312) | 4.3% (192) |
| No physical disability | 93.7% (7,534) | 91.2% (3,249) | 95.7% (4,285) |
| No answer | 0.04% (2) | 0.03% (1) | 0.04% (2) |
|  |  |  |  |
|  | **Total households (N=2,587)** |  |  |
|  | % (n) |  |  |
| **Household size** |  |  |  |
| 1-5 members | 33.9% (877) |  |  |
| 6-10 members | 57.1% (1,476) |  |  |
| 11-25 members | 9.1% (234) |  |  |
| Mean No. of household members (SD) | 6.8 (2.9) |  |  |
| **Monthly household income** (N=2,192) |  |  |  |
| Lowest (first quintile) | 23.2% (509) |  |  |
| (2nd quintile) | 28.7% (629) |  |  |
| (3rd quintile) | 13.3% (292) |  |  |
| (4th quintile) | 18.2% (399) |  |  |
| Highest (5th quintile) | 16.6% (363) |  |  |
| Mean income (SD) in USD | 338.3 (256.4) |  |  |
| Median income (IQR) in USD | 282.9 (198.1, 424.5) |  |  |
| **Monthly household expenditures** (N=2,287) |  |  |  |
| Mean expenditures (SD) in USD | 508.2 (318.5) |  |  |
| Median expenditures (IQR) in USD | 424.5 (353.7, 565.9) |  |  |
| **Household debt** |  |  |  |
| Household in debt | 79.3% (2,052) |  |  |
| No debt | 20.0% (516) |  |  |
| No answer | 0.7% (19) |  |  |
| **WFP food vouchers** |  |  |  |
| Household received food vouchers | 92.9% (2,404) |  |  |
| **Location of household** |  |  |  |
| Rural | 45.3% (1,171) |  |  |
| Urban | 54.7% (1,416) |  |  |
