# Supplementary material for: Prevalence of non-communicable diseases and access to care among non-camp Syrian refugees in northern Jordan
Source: Confl Health. 2018 Jul 11;12:33. doi: 10.1186/s13031-018-0168-7 (PMC6040066; doi:10.1186/s13031-018-0168-7)
Supplement: Supplementary file 2 — Table S2. Most frequently reported single- and multi-morbidities among adults with at least one NCD. (N = 1756). (DOCX 14 kb) [file 13031_2018_168_MOESM2_ESM.docx]

**Additional File 2**

**Table 2: Most frequently reported single- and multi-morbidities among adults with at least one NCD.** (N=1,756)

| **Single- and multi-morbidities** | **n** | **% (95%CI)** |
| --- | --- | --- |
| Hypertension only | 399 | 22.7% (20.8-24.8) |
| Diabetes & hypertension | 309 | 17.6% (15.9-19.5) |
| Diabetes only | 178 | 10.1% (8.7-11.8) |
| Respiratory diseases only | 160 | 9.1% (7.8-10.6) |
| Diabetes, hypertension & CVD | 143 | 8.1% (6.9-9.7) |
| Hypertension & CVD | 124 | 7.1% (5.9-8.4) |
| Thyroid disease only | 111 | 6.3% (5.3-7.5) |
| CVD only | 102 | 5.8% (4.8-7.0) |
| Diabetes, hypertension & thyroid disease | 23 | 1.3% (0.9-2.0) |
| Hypertension & chronic respiratory conditions | 22 | 1.3% (0.8-1.9) |
